# The Optimal Health Weight and Lifestyle (OHWL) Clinic: Comprehensive, Multidisciplinary and Geriatric-Focused Care for Multimorbid Older Adults With Obesity

**DOI:** 10.1016/j.cdnut.2025.107526

**Published:** 2025-08-14

**Authors:** Shenbagam Dewar, Mary R Janevic, John A Batsis, Neil B Alexander

**Affiliations:** 1 Division of Geriatric and Palliative Medicine, Department of Internal Medicine, University of Michigan Medical School, Ann Arbor, MI, United States; 2 Geriatric Research Education and Clinical Center (GRECC), Veterans Affairs Ann Arbor Healthcare system, Ann Arbor, MI, United States; 3 Department of Health Behavior and Health Equity, School of Public Health, University of Michigan, Ann Arbor, MI, United States; 4 Department of Nutrition, Gillings School of Global Public Health, University of North Carolina at Chapel Hill, Chapel Hill, NC, United States; 5 Division of Geriatric Medicine, UNC School of Medicine, Chapel Hill, NC, United States

**Keywords:** polypharmacy, pain interference, grip strength, sarcopenia, caregiver dependency, hand dynamometer, area deprivation index, incretin mimetics, restless legs syndrome

## Abstract

**Background:**

The prevalence of obesity among older adults is increasing, and care is complicated by comorbidities.

**Objectives:**

This study describes the baseline cohort of the Optimal Health, Weight, and Lifestyle clinic, designed to address weight management using geriatric principles with focus on comorbidities, function, and quality of life. It also highlights the need for multidisciplinary, coordinated care for this population.

**Methods:**

This is a retrospective study of patients (*n* = 58) with a body mass index (BMI) ≥ 30 kg/m^2^ referred for weight management. Assessments included obesity-related comorbidities; self-reported measures for pain, physical health, and mental health using Patient-Reported Outcomes Measurement Information System (PROMIS) scores; grip strength using an in-clinic dynamometer; and specialist care.

**Results:**

The mean (standard deviation) age of the 58 patients was 73.4 (4.8) y, and they were predominantly female (76%) and White (81%). With a mean of over 12 comorbidities, common comorbidities included chronic kidney disease (98%), hypertension (97%), osteoarthritis (93%), obstructive sleep apnea (83%), prediabetes (42%), and diabetes (42%). Patients were comanaged by an average of 4 specialists. PROMIS physical and mental health scores were low at 40 and 45 y, respectively, and worsened with increasing BMI. Thirty-six percent had physical activity limitations, and 30% were dissatisfied with social activities and relationships. Grip strength was below age/gender norms in most patients.

**Conclusions:**

Older adults with obesity have multiple comorbidities, low strength, and high pain interference, and receive care from multiple specialists, placing them at risk of fragmented care. A person-centered, geriatric-focused approach incorporating the Medication, Mentation, Mobility, and What Matters (4Ms) framework is needed, along with comprehensive obesity care provided by a team-based approach. Further evidence-based interventions adapting lifestyle and medication management to this cohort are also needed.

## Introduction

Aging with obesity leads to significant multimorbidity and an increased risk of functional decline [1], decreased quality of life, institutionalization [2], and increased mortality [3]. Older adults experience an accelerated worsening of health conditions, impacting health related to osteoarthritis [4], cardiopulmonary disorders [5], diabetes, and liver and kidney disease [6]. With the prevalence of obesity in older adults estimated at 39% [7], aging with obesity exerts a major influence on health care across all settings. Obesity compromises healthy aging across 5 key domains—cognitive health, physical health, mental health, chronic disease management, and social engagement—by increasing the early onset of chronic disease, functional limitations, and cognitive decline. Although the Institute for Healthcare Improvement advocates for age-friendly care delivery for older adults across all settings, the Obesity and Equitable Aging Group calls for comprehensive, system-wide reforms to establish equitable strategies for the prevention and management of obesity in older adults [8]. These highlight the critical need for age-friendly care tailored to older adults with obesity, utilizing the Medication, Mentation, Mobility, and What Matters (4Ms) framework, as this population demonstrates features of accelerated physiological aging and faces heightened vulnerability to adverse health outcomes.

Obesity is one of the most undertreated chronic conditions [9], and primary care physicians have been particularly reluctant to treat older adults for weight loss, both in the community and residential settings, due to concerns about exacerbating age-related muscle and bone loss [10]. Current weight loss recommendations include behavioral and lifestyle interventions (to improve health behaviors such as diet and physical activity), prescription of obesity medications (OMs), and bariatric surgery [11].

Engagement in lifestyle interventions is variable among older adults, often due to low levels of calorie knowledge, low calorie confidence, and physical exhaustion due to medical conditions [12,13]. This is further worsened by social barriers, such as a less-advantaged home microenvironment, limited access to urban neighborhoods that promote physical activity, and higher food insecurity among older adults [14,15]. Multimorbidity leads to worsening functional impairment, mobility limitations, and disability. These challenges make lifestyle interventions less likely to be emphasized by clinicians and less likely to be adopted by patients. Clinicians currently providing care for older adults face mounting pressure to treat obesity with the new emerging incretin mimetics, although still lacking long-term outcome data. This poses a risk to a subset of older adults due to inappropriate patient selection for treatment with incretin mimetics, age-insensitive titration leading to adverse effects, and less-desired long-term outcomes on muscle and bone, further emphasizing the need for an age-friendly approach to obesity care.

There is thus an urgent need to characterize the vulnerable population of older adults with obesity and multimorbidity. Clinical practice guidelines for weight management in older adults with multimorbidity and functional impairment remain limited, underscoring the need for an age-friendly approach that carefully integrates patient health priorities, physical and mental health status, mobility limitations, and pharmacological considerations, with particular emphasis on the deprescribing of medications that may contribute to obesity [16]. Hence, careful patient selection is therefore of utmost importance, and accordingly, we developed a new outpatient model, the “Optimal Health Weight Lifestyle” (OHWL) clinic, led by an obesity- and geriatric medicine–trained physician. Currently, few data exist on clinical profiles and patient-reported outcomes in older adults with obesity and multimorbidity. To fill this gap, the present study reports on the baseline characteristics of the initial OHWL clinic cohort, including the prevalence of multimorbidity, physical strength, pain, and how these factors relate to BMI (in kg/m^2^). We hypothesized that this cohort, with increasing BMI, would exhibit high levels of multimorbidity and physical- and mental health–related symptoms. This real-world study provides insight into the medical complexity as well as functional and psychosocial impairments in this cohort, highlighting the need for a team-based model of care within an age-friendly approach across both health care and residential settings.

### Subjects

Older adult patients (age ≥ 60 y) were referred for weight management with a goal to optimize obesity-related multimorbid conditions. Eligibility criteria included BMI ≥ 30, ability to walk across a room with or without an assistive device, and living in the community or residential settings but not in a long-term care nursing home (with or without caregiver assistance). Participants were required to have ≥2 obesity-related comorbidities. Patients were excluded if they had moderate cognitive impairment (Montreal Cognitive Assessment score < 18) [17] or major/unstable psychiatric illness as determined by the referring providers.

## Methods

### Clinic setting

Led by an obesity- and geriatric medicine–trained physician, the OHWL clinic is colocated within an academic geriatric primary care clinic setting. The goal of the clinic is to promote slow and tailored weight loss although improving lifestyle and overall health: *1*) optimizing care of obesity-related conditions and comanagement with specialists on advanced comorbid disease conditions; *2*) improving mobility, by referrals to physical therapy to promote physical activity; *3*) diet modification, by referrals to dietitian; and *4*) pharmacotherapy in select patients with an age-friendly approach.

Data were collected from the first 58 patients referred to the clinic from November 2018 to March 2022. The majority of patients were referred from geriatric primary care (88%), followed by mobility specialists (5.2%). The remaining patients were referred by adult primary care providers, both within and outside the health system, as well as by cardiology and through self-referral. This study was approved by the University of Michigan Institutional Review Board (HUM00199706).

### Electronic health record–based assessment

Participants completed previsit questionnaires, including the PROMIS-10 Global Health scale, assessing physical health status (including overall health rating and physical functioning) and mental health status (including overall mental health rating, mood problems, and satisfaction with social activities and relationships). Health status scores were converted to T-scores. Cutoffs for “low” scores indicating fair to poor health were used (Global Physical Health: T-score < 42 and Global Mental Health: T-score < 40). A subanalysis of individual item scores for physical functioning and satisfaction with social activities and relationships was utilized to assess self-report on function and social connection domains. In addition, T-score conversions of raw summations of the PROMIS Pain Interference (Short Form 6A) items included pain interference with daily activities, such as household chores and social activities in the last 7 d (1 = not at all to 5 = very much).

Gender and race were determined from the electronic health record (EHR). The area deprivation index (ADI) was determined from the Neighborhood Atlas [mapping (wisc.edu)] based on each patient's home address. The ADI [18] provides a percentile ranking of neighborhoods by socioeconomic disadvantage (at both the state and national levels) with (state; range: 1–10) and (national; range: 8–99) higher scores indicating more disadvantaged neighborhood.

### Clinic and EHR assessment

With measured height and weight information at intake by a medical assistant, obesity-related BMI categories were determined: class 1 obesity (BMI: 30–34.9), class 2 obesity (BMI: 35–39.9), class 3 obesity (BMI ≥ 40–49.9), and classes 4 and 5 obesity (BMI > 50). Waist circumference was measured by the medical assistant using the National Heart, Lung, and Blood Institute protocol, with the measurement taken directly above the uppermost border of the iliac crest.

During the in-person visit, the EHR was reviewed for key obesity-relevant conditions and confirmed with the patient: metabolic diseases; cardiovascular diseases; chronic kidney disease (CKD); obstructive sleep apnea and other obesity-related sleep disorders; chronic obstructive pulmonary disease/asthma; edema, particularly lymphedema; liver disease; osteoarthritis; chronic pain; and cancer (both obesity related [19] and unrelated). Also included were commonly prevalent mental health disorders in adults with obesity.

Metabolic syndrome was defined by the National Cholesterol Education Program [20]. Risk of H_2_FpEF [21] was computed using clinical and echocardiogram characteristics (score: low 0–1, intermediate 2–5, and high 6–9). Known atherosclerotic cardiovascular disease (ASCVD) was defined as a history of coronary artery disease, cerebrovascular accident (stroke or transient ischemic attack), or peripheral arterial disease. Ten-year risk of acquiring ASCVD was estimated from the American College of Cardiology/American Heart Association calculator [22]: low (<5%), borderline (5%–7.4%), intermediate (7.5%–19.9%), and high (>20%).

Patients were asked to report the presence or the absence of pain (including musculoskeletal and neuropathic pain). We calculated participants’ number of comorbid health conditions, out of 17 obesity-related comorbidities.

The number of specialists involved in the comanagement, as part of the clinical care team at the initial visit, was determined by tracking encounters in the EHR from the health system–based clinics; additional tracking expanded to outside the health system, via the health information exchange, and these data were further confirmed with the patient.

Those who received assistance with ≥1 mobility, self-care, or household task for health and functioning reasons were asked to identify their need for caregiver(s). Participants were asked to report on the use of an assistive device for mobility (cane, walker/rollator, or wheelchair/scooter). We assessed left and right hand grip strength, shown to predict limb strength and mobility disability [23], using a handheld dynamometer (JAMAR; JLW Instruments). This was measured in both hands 3 times, alternating every 30 s, and the highest value was used in the calculation.

### Statistical analysis

All analyses were completed using IBM SPSS Statistics, v. 28.0.0.0. Descriptive statistics (means and frequencies) were calculated for key demographic, biometric, clinical, and patient-reported variables. To assess the relationship of BMI class with physical and psychosocial functioning, 1-way analysis of variance was used for continuous outcome variables (PROMIS variables), Pearson’s *χ*^2^ tests were used for categorical outcomes (need for caregiver), and Pearson’s *r* was used for correlations. A *P* value of 0.05 was considered statistically significant.

## Results

### Sample characteristics

Fifty-eight patients had a mean (SD) age of 73.4 (4.8) y and were predominantly female (76%) and White (81%) (Table 1). They were covered predominantly by Medicare Advantage or Medicare with supplemental plans and lived in the most advantaged third to half neighborhoods on the state and national deprivation indices. All patients lived in the community setting except 2, who lived in adult foster home and assisted living (not shown in the table). The mean (SD) BMI was 41.3 (7.5), range of 30.3–64.4, and obesity class distribution was as follows: class 1 (BMI = 30–34.9; *n* = 13, 22%), class 2 (BMI = 35–39.9; *n* =17, 29%), class 3 (BMI = 40–49.9; *n* = 20, 34%), class 4 (BMI = 50–59.9; *n* = 7, 12%), and class 5 (BMI > 60; *n* = 1, 2%).

**TABLE 1:** OHWL cohort sample demographics and Medical Data (*N* = 58 unless otherwise noted).

	Mean (SD) or % (*n*)
Age (y; range 60–83)	73.4 (4.8)
Female	76% (44)
Race	
African American/Black	13.8% (8)
White	81.0% (47)
Other	5.2% (3)
Insurance plan	
Medicare only	1.7% (1)
Medicare advantage	43.1 (25)
Medicare with supplement	43.1 (25)
Medicare and VA	3.4 (2)
Medicare and Medicaid	8.6% (5)
Referral source	
Geriatric primary care	88% (51)
Primary care (within health system)	1.7% (1)
Primary care (outside health system)	1.7% (1)
Mobility specialist	5.2% (3)
Cardiology	1.7% (1)
Self-referred	1.7% (1)
Deprivation index (state; range: 1–10)	2.8 (2.5)
Deprivation index (national; range: 8–99)	41.1 (23.7)
Has caregiver (*n* = 56)	24.1% (14)
BMI (range: 30.3–64.4), kg/m^2^	41.3 (7.5)
BMI obesity class	
1: 30–34.9	22.4% (13)
2: 35–39.9	29.3% (17)
3a: 40–49.9	34.5% (20)
3b: 50+	13.8% (8)
Waist circumference (cm; range: 99.1–147.3) (*n* = 48)	124.3 (13.0)
Hand grip strength (kg)	
Right hand (range: 9–50)	23.4. (10.6)
Right hand deviation from age/gender norm^1^	6.2 (4.4)
Left hand (range 5–45)	21.2 (9.8)
Left hand deviation from age/gender norm^1^	6.0 (4.1)

Abbreviation: VA, Veterans Affairs.

1 Calculated by subtracting grip strength from age/gender norm, such that a positive deviation indicated grip strength below the norm.

### Comorbid medical conditions

With a mean number of comorbidities exceeding 12.1 (SD: 1.9), almost all participants had hypertension (97%) and osteoarthritis (93%). More than three-quarters (78%) met criteria for metabolic syndrome, and nearly all had prediabetes or diabetes mellitus (41% each) (Table 2). Thirteen participants (22%) were diagnosed with heart failure, all but 1 with heart failure with preserved ejection fraction (HFpEF). Excluding these 13, 29 patients had echocardiograms and then a computed H_2_FpEF mean (SD) 4.9 (1.4) score. Based on H_2_FpEF score, 17% of these 29 patients were noted to be at a high risk and 83% at an intermediate risk of HFpEF. Sixteen of the 58 (28%) patients had known ASCVD. In those without known ASCVD (*n* = 40), the mean 10-y ASCVD risk score was 23.9, in the high range, with 45% scoring in the high-risk (>20%) category. CKD was highly prevalent, affecting 98% of the participants; additionally, 50% had stage 3 or more advanced CKD. Approximately three-fourths of the cohort had elevated liver enzymes, and 33% were diagnosed with metabolic dysfunction–associated steatotic liver disease (MASLD) based on imaging. Sleep disorders were common, including obstructive sleep apnea (83%), insomnia (67%), snoring (53%), and restless leg syndrome (22%). Major depression and anxiety disorders were the most common mental health diagnoses.

**TABLE 2:** Prevalence of comorbid medical conditions in the study cohort.

Conditions	Mean (SD) or % (*n*)
Chronic kidney disease (GFR categories)	98% (57)
G1: Normal or high >90	1.7% (1)
G2: Mildly decreased 60–89	48.3% (28)
G3 or below: Mildly to moderately decreased to severely decreased 15–59	50% (29)
Hypertension	96.6% (56)
Chronic pain	94.8% (55)
Osteoarthritis	93.1% (54)
Obstructive sleep apnea	82.8% (48)
Dyslipidemia (*n* = 56)	79.3% (46)
Mental health disorders	79.3% (46)
Metabolic syndrome	77.6% (45)
Hyperuricemia	67.2% (39)
Other sleep disorders	
Insomnia	67.2% (39)
Primary snoring	53.4% (31)
Restless Legs Syndrome	22.4% (13)
Asthma or COPD	55.2% (32)
Glycemic status	
Diabetes	41.4% (24)
Prediabetes	41.4% (24)
Normal	17.2% (10)
Heart failure	22.4% (13)
Atrial fibrillation	19% (11)
Leg edema	60.3% (35)
Venous stasis	31.0% (18)
Lymphedema	27.6% (16)
Any cancer	24.1% (14)
Obesity-related	13.8% (8)
Nonobesity related^1^	13.8% (6)
Liver dysfunction	
Normal LFT	24.1% (14)
Elevated LFT	75.9% (44)
MASLD by US	32.8% (19)
H_2_FPEF risk score categories (*n* = 29)	
Low (0–1)	0% (0)
Intermediate (2–5)	82.8% (24)
High (6–9)	17.2% (5)
% of those with history of ASCVD (*n* = 16)	28%
Mean (SD) ASCVD risk score (10-y % risk in those without ASCVD, *n* = 40, range: 6.4–61.5)	24.0 (13.6) %
% With high-risk ASCVD (>20% 10-y risk) (18/40)	45%

H_2_FPEF risk score variable excludes patients without an echocardiogram to compute score (n=16) and those with established heart failure (n=13) to compute score for the remaining 29.

Abbreviations: ASCVD, atherosclerotic cardiovascular disease; COPD, chronic obstructive pulmonary disease; G1, G2 and G3, stages of kidney disease; GFR, glomerular filtration rate; H_2_FPEF, heart failure with preserved ejection fraction; LFT, liver function test; MASLD, metabolic dysfunction–associated steatotic liver disease; US, ultrasonography.

Count of comorbid conditions in Table 2 includes**:** Hypertension, atrial fibrillation, diabetes or prediabetes, dyslipidemia, metabolic syndrome, obstructive sleep apnea, other sleep disorder (insomnia, snoring, and/or restless legs syndrome), coronary artery disease/stroke OR ASCVD score > 7.5, congestive heart failure OR HFpEF score that is intermediate OR greater, chronic kidney disease (eGFR <60), hyperuricemia, asthma or chronic obstructive pulmonary disease, lower extremity swelling (leg edema, venous stasis, and/or lymphedema), liver dysfunction (elevated LFT or MASLD by US), cancer, mental health disorder (major depression, bipolar disorder, panic disorder, other anxiety disorder, post-traumatic stress disorder, personality disorder, eating disorder, and/or alcohol use disorder) and osteoarthritis.

1 Non–obesity-related cancer in this analysis includes cervical cancer, vulval intraepithelial neoplasia III, basal cell carcinoma, malignant melanoma (2) and prostate cancer.

**FIGURE 1:**
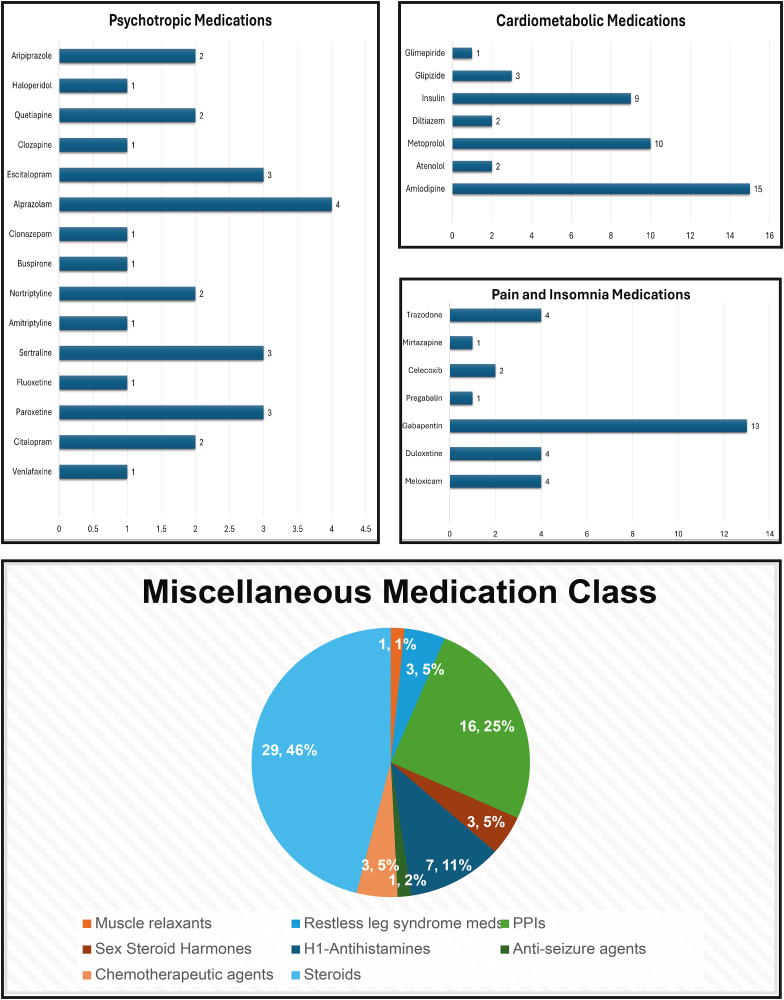
Distribution of obesogenic medications by class among participants in the pilot cohort (*n* = 58). Bar graphs depict the frequency of individual obesogenic medications prescribed within 3 major classes: psychotropic medications, cardiometabolic medications, and pain/insomnia medications. A pie chart illustrates the proportional distribution of additional obesogenic medication classes designated as “Miscellaneous,” including muscle relaxants (baclofen), sex steroid hormones (estrogen, testosterone), chemotherapeutic agents (anastrozole, letrozole), restless leg syndrome medications (pramipexole), H1-antihistamines (cetirizine), steroids (oral, inhaled, and nasal), proton pump inhibitors (omeprazole, esomeprazole, lansoprazole), and antiseizure agent (valproic acid). Values represent the number of individuals in the cohort prescribed each medication or medication class. PPI, Proton Pump Inhibitor.

Participants were prescribed a mean of 16 medications (range: 4–31), including a mean of 3 obesogenic medications (range: 0–8; Figure 1). Most patients reported pain (95%), and 79% used pain medications. The pain medications included nonopioid analgesics, including acetaminophen, nonsteroidal anti-inflammatory drugs, opioids, neuropathic pain medications (gabapentin and lyrica), dopamine agonists (pramipexole and ropinirole) for pain due to restless leg syndrome, and topical pain medications. Weight-promoting medications in our cohort primarily included psychotropic agents prescribed for anxiety and depression, antihypertensive and hypoglycemic agents used for the management of cardiometabolic conditions, as well as medications for neuropathic pain (Figure 1).

### Specialty care utilization

This highly comorbid cohort was comanaged by an average of 4.2 different medical specialists as part of the clinical care team at the time of the initial assessment; with 74% of the patients consulting ≥3 specialists (Table 3). The 5 most commonly consulted specialists were of sleep medicine (60%), cardiology/heart failure (43%), orthopedics (38%), surgery (38%), and endocrinology (35%).

**TABLE 3:** Specialists involved in clinical care teams at the time of OHWL clinic enrollment (*n* = 58 patients).

Mean (SD) specialists	4.2 (2.4)
Number of specialists % (*n*)	
No specialist	3.4% (2)
1−2 specialists per patient	22.4% (13)
3+ specialists per patient	74.2% (43)
Type of specialist referrals in past year % (*n*)	
Sleep medicine	60.3% (35)
Cardiology/heart failure	43.1% (25)
Orthopedics	37.9% (22)
Surgery	37.9% (22)
Endocrinology	34.5% (20)
Gastroenterology	27.6% (16)
Physical medicine and rehabilitation	25.9% (15)
Psychiatry	22.4% (13)
Pulmonary	15.5% (9)
Pain medicine	15.5% (9)
Urology	13.8% (8)
Neurology	13.8% (8)
Oncology	12.1% (7)
Podiatry	10.3% (6)
Hepatology	6.9% (4)
Lymphedema	1.7% (1)
Other specialist(s)	29.3% (17)

### Quality of life

The PROMIS Global-10 Physical Health [mean (SD)]: [40 (9)] and Mental Health [45 (8)] summary T-scores were 1 and 0.5 SDs lower (indicating worse health) than the population mean, respectively. The Pain Interference summary T-score [58 (9)] was ∼1 SD higher (indicating more pain), than the population mean. Of note, higher pain interference was highly correlated with lower physical (*r* = −0.68, *P* < 0.001) and mental (*r* = −0.42, *P* = 0.01) health scores (not shown in the table). Among those who completed the PROMIS-10 Global score questionnaire, 36% were unable or had only little ability to carry out everyday physical activities, and 30% showed fair to poor satisfaction with social activities and relationships (social connection) (not shown in the table).

### Relationship of BMI class to physical and psychosocial functioning

Pain interference T-scores increased with BMI class (*P* = 0.006), such that scores in classes 4 and 5 obesity were nearly 16 points greater than those in class 1 obesity, representing a difference between 1 and 2 SDs in this population-normed score (Table 4). On the other hand, although low PROMIS Physical Health scores were more common with increased BMI class, the main difference occurred between class 1 obesity and the other classes (*P* = 0.009). Nearly three-quarters of patients with classes 4 and 5 obesity had low mental health scores and required a caregiver to support daily function.

**TABLE 4:** Association of obesity class with patient functioning.

	BMI 30–34.99	BMI 35–39.99	BMI 40–49.99	BMI 50+	*P* value
	Mean (SD) or % (*n*)				
PROMIS Physical Health – low score	27.3% (3)	82.4% (14)	47.1% (8)	85.7% (6)	0.009
PROMIS Mental Health – low score	18.2% (2)	23.5% (4)	17.6% (3)	71.4% (5)	0.040
PROMIS Pain Interference score	52.4 (8.6)	56.2 (7.1)	60.1 (5.4)	68.0 (10.2)	0.006
Has caregiver	7.7% (1)	18.8% (3)	25.0% (5)	71.4% (5)	0.015

Abbreviation: PROMIS, Patient-Reported Outcomes Measurement Information System.

### Grip strength

Of the 38 participants with available grip strength data, mean (SD) grip strength (in kilograms) was 23 (11) kg and 21 (4) kg for the right and left hand, respectively. These values were below age/gender norms [24] in 31 and 28 participants, respectively, by a mean (SD) 6 (4) kg on both right and left sides (Table 1).

### Relationship of BMI class to assistive device dependency

**FIGURE 2:**
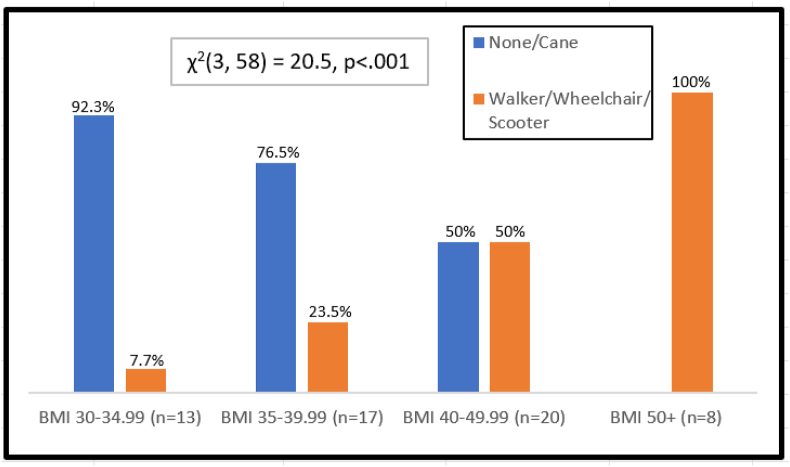
Type of assistive device use by BMI class. The percentage of individuals using none/cane versus walker/wheelchair/scooter is shown for each BMI group. Higher BMI class is associated with greater use of walkers, wheelchairs, or scooters [*χ*^2^(3, 58) = 20.5, *P* < 0.001].

Assistive device dependency increased with BMI, and 50% of the patients with BMI > 50 used a walker or a rollator, and the remaining 50% used a wheelchair or a scooter for mobility (Figure 2).

## Discussion

This real-world study highlights the substantial burden of multimorbidity observed in our cohort, with a mean comorbidity count of 12.1 (SD: 1.9) as assessed by the clinical care team and confirmed by diagnostic data. This figure far exceeds the average comorbidity count typically reported in analyses based on insurance claims data from the general population aged > 60 y, using International Classification of Diseases, 10th Revision diagnosis codes [25]. In comparison, a similar clinic-based study of older adults in Brazil (mean age: 65 y; mean BMI: 38.5) reported a median of 7 diagnoses, many of which were also prevalent in our cohort [26]. The higher mean age (73 y), greater mean BMI (41.3), and referral criteria requiring ≥2 obesity-related comorbidities in our study likely contributed to the greater degree of multimorbidity observed. Our findings indicate that advancing age combined with obesity is linked to a greater burden of multimorbidity with significant clinical implications. The psychiatric disorders, pain, mobility, and functional impairment observed among this subgroup may explain the degree of psychosocial impairment and caregiver dependency. All these mentioned conditions likely contribute to the low quality of life scores observed, especially at the highest level of obesity.

Among the comorbid conditions, obstructive sleep apnea, osteoarthritis, cardiometabolic diseases, and CKD were the most common. Significant and concerning was the high prevalence of HFpEF and the risk for future HFpEF and ASCVD observed among the patients. The risk for heart failure increases 5% in men and 7% in females for each 1 increment in BMI. This finding underscores the importance of systematic screening for HFpEF as well as the need for early detection and intervention strategies. In this study, we utilized the H_2_FPEF screening score, which incorporates both clinical and echocardiographic parameters. Key factors such as obesity (heavy), hypertension, atrial fibrillation, pulmonary hypertension, advanced age (elderly), and elevated heart filling pressures are integrated into the scoring system to assess the likelihood of HFpEF by assigning a numerical value to each risk component (Table 2). Targeting modest weight loss (5%) by caloric restriction and pharmacotherapy can result in functional improvement in patients with HFpEF [27]. The semaglutide effects on cardiovascular outcomes in people with overweight or obesity (SELECT) cardiovascular outcome trial found that the use of a semaglutide led to 20% reduction in the incidence of death from cardiovascular causes, nonfatal myocardial infarction, or nonfatal stroke among adults older than 45 y [28]. The Semaglutide Upon Major Morbidity and In Terminal illness (SUMMIT) phase 3 trial showed that semaglutide reduced the risk of heart failure outcomes by 38% and also reduction in heart failure urgent visit or hospitalization, oral diuretic intensification, heart failure symptoms, physical limitations, or cardiovascular death. It also helped achieve 15.7% weight loss in a combined population of adults with and without type 2 diabetes [29]. The high prevalence of CKD (50%) in the present study highlights the need to target weight loss, because behavioral, pharmacological, and bariatric surgical intervention have been associated with slowing glomerular filtration rate decline [30]. The Finding the efficacy of semagLutide in chronic kidney disease Outing for patients with type 2 diabetes Worldwide (FLOW) trial demonstrated that a semaglutide at a lower dose of 1 mg weekly reduced the risk of clinically important kidney outcomes by 24% while slowing the chronic disease progression [31]. In addition, the high prevalence of abnormal liver enzymes (76%) and MASLD (33%) in this cohort is concerning, particularly given the rising rates of hepatobiliary cancers among older adults (70–79 y of age) associated with obesity, metabolic syndrome, and traditional risk factors such as chronic hepatitis and alcoholic cirrhosis [32]. Bariatric surgery and newer OMs are emerging as promising treatment options as noted in the Veterans Health administration study on glucagon-like polypeptide-1 agents and pemvidutide clinical trial [33,34].

The high number of comorbidities, specifically related to obesity and in advanced stages, leads to multispecialty care, as seen in this cohort, with a mean of over 4 different specialists involved, contributing to the increased ambulatory health care cost and risk for care fragmentation. This also contributes to the polypharmacy burden observed in this cohort, which is further complicated by the use of weight-promoting medications. These findings underscore the importance of the clinical care team conducting thorough medication reviews and implementing deprescribing strategies when appropriate. This warrants further emphasis as this cohort—with mobility impairment and evidence of reduced skeletal muscle function as indicated by below-normal hand grip strength—had a high prevalence of pain medication prescriptions (∼80%). This in the context of polypharmacy may increase the risk of falls and delirium. The addition of incretin-mimetic prescriptions to promote weight loss, in the context of polypharmacy and reduced muscle function, may further exacerbate the risk of falls due to potential unintended effects on muscle.

The heightened multimorbidity and complexity of treatment plans developed by multiple specialists necessitate coordinated care by the primary care physician and the health care team, recognizing that underlying obesity is the primary driver of advanced comorbid conditions. This further emphasizes the need to reinforce counseling for lifestyle intervention and other modalities for effective weight loss while comanaging with specialists, as obesity-related medical conditions tend to worsen. A team-based model of care facilitated by a care manager as warranted in other geriatric conditions such as dementia and heart failure is thus likely needed for obesity as it is recognized as a chronic disease (American Medical Association, 2013) [35]. Due to the neurobehavioral and psychosocial underpinning of the condition and prevalence of social determinants of health (SDOH), a social worker and health coach could play a pivotal role with the interdisciplinary team along with specialist comanagement.

Grip strength was below age/gender norms in most of the participants. Using criteria for abdominal obesity (waist circumference of >88 cm in females and >102 cm in males) and dynapenia (hand grip weakness of <20 kg in females and <30 kg in males), 1 male and 20 females (21/38 or 55%) met criteria for dynapenic abdominal obesity, which is thought to be predictive of adverse outcomes such as reduced mobility.

The dependency on advanced mobility devices increased with higher BMI as anticipated. This has an enormous impact on the overall obesity care plans and suggests a need for an age-friendly approach to care with team-based care coordination. This vulnerable population is thus at risk for caregiver dependency and is at risk of transitioning to a more supportive living environment.

Newer anti-OMs, particularly incretin mimetics, serve as adjuncts in obesity management, as demonstrated by the cardiovascular benefits observed in the clinical trial program evaluating the effects of semaglutide in people with type 2 diabetes (SUSTAIN-6) and Liraglutide Effect and Action in DiabetEs: Evaluation of cardiovascular outcome Results (LEADER) clinical trials [36,37]. However, these agents were associated with increased reports of gastrointestinal side effects, and semaglutide was linked to a higher incidence of diabetic retinopathy events. There is concern that tirzepatide and semaglutide may contribute to the loss of ≤25% and 39%, respectively, of lean body mass as observed during clinical trials. Despite demonstrated short- and intermediate-term efficacy, data on long-term safety and efficacy in older adults, including effects on muscle and bone loss as well as changes in functional status, remain limited [38]. Although awaiting future randomized trials, an updated algorithm based on current evidence in 2024 recommends pharmacological management with newer generation OMs (semaglutide, liraglutide, and tirzepatide) or orlistat and phentermine–topiramate after trial of semaglutide, for BMI >30 or ≥27 with cardiometabolic comorbidity and refractory to lifestyle intervention [39].

Higher BMI, waist circumference, multimorbidity, and mobility impairment with hand grip strength below age- and sex-specific norms in most participants suggest an elevated risk of sarcopenia and predisposition to frailty in this study population. These findings underscore the potential for iatrogenic loss of muscle and bone mass if weight loss is pursued without a comprehensive geriatric assessment. This would include assessment for cognition, activities of daily living (ADL), and instrumental ADL impairment, strength, mobility, and balance assessment (handgrip strength, chair stand, and timed up and go test), psychosocial assessment using Patient Health Questionnaire-2 screen, and SDOH screening for housing, food insecurity, and transportation [16]. The risk for muscle and bone loss is further exaggerated with the addition of newer incretin mimetics to the management algorithm. This underscores the importance of a comprehensive weight loss program with an emphasis on protein, micronutrients, and physical activity, with specific focus on resistance training [40].

### Study limitations

Given that patients were drawn from an academic geriatric primary care clinic, the patient sample is from an advantaged neighborhood and less representative of the general population. Origin of the referrals from geriatric primary care and heart failure clinic with an eligibility criteria including 2 obesity-related comorbidities might have skewed the sample toward greater multimorbidity and poorer overall health. The predominance of Whites in this study limits racial diversity, but the higher proportion of females is not uncommon in studies relating to obesity as females seek obesity interventions. The accuracy and completeness of the comorbidities and the specialists are limited by the breadth of the health information exchange, despite crosschecking with the patient at the initial encounter.

In conclusion, adults with obesity have multiple comorbidities, low strength, and high pain interference with daily activities; receive care from multiple specialists; and hence remain at risk for fragmented care. An age-friendly 4Ms approach with a person-centered, geriatric focused, and comprehensive obesity care with a team-based approach is needed in this population. Further evidence-based interventions, adapting lifestyle interventions, and OM management to this cohort are also needed.

## Author contributions

All listed authors contributed to the study design and manuscript writing had full access to all the data in the study, take responsibility for the integrity of the data and the accuracy of the data analysis, and had authority over manuscript preparation, the decision to submit the manuscript for publication, and approved its current contents. All authors meet the criteria for authorship stated in the Uniform Requirements for Manuscripts Submitted to Biomedical Journals.

## Funding

The authors reported no funding received for this study.

## Conflict of interest

John Batsis is a guest editor for *Current Developments in Nutrition* and played no role in the Journal's evaluation of the manuscript
